# War diseases revealed by the social media: massive leishmaniasis outbreak in the Syrian Spring

**DOI:** 10.1186/1756-3305-6-94

**Published:** 2013-04-12

**Authors:** Samer Alasaad

**Affiliations:** 1 Department of Ecology and Sylviculture, Euphrates University, Deir Ezzor, Syria; 2 Institute of Evolutionary Biology and Environmental Studies (IEU), University of Zürich, Zürich, Switzerland; 3 Estación Biológica de Doñana, Consejo Superior de Investigaciones Científicas (CSIC), Sevilla, Spain

**Keywords:** Social Media, Facebook, YouTube, Syrian Arab Republic, Cutaneous leishmaniasis, *Leishmania tropica* complex, Arab Spring, Revolution, Deir Ezzor

## Abstract

Social media introduce pivotal changes to communication between individuals, organizations and communities. A clear example of the power of social media is the spread of the revolutionary outbreaks in the Arabic countries during 2011, where people used Facebook, YouTube and Skype to communicate, organise meetings and protest actions. Here I report how Doctor-Activists use these social media as an alarm system for ‘war disease’ outbreaks in the Syrian Spring. Social media are used as an alarm system to attract the attention of international organizations, which should assume their responsibilities and play their part in controlling the outbreak of such war diseases.

## 

Social media are usually defined as the means of interactions in which people create, share and exchange ideas and information based on virtual communities and networks [1]. Nowadays, mobile and web-based technologies are the motor of social media, in which people create interactive platforms where they discuss, co-create, share and modify content with a user-generated character. In this way, social media introduce pivotal changes to communication between individuals, organizations and communities [2].

Despite some criticism of social media [3], they have mainly positive effects: learn and explore, advertise oneself, form friendships and even document memories. A clear example of the power of social media is the spread of the revolutionary outbreaks in the Arabic World during 2011, where people used Facebook, YouTube and Skype to demand freedom, organise meetings, protest actions *etc.*[4,5].

Before the Syrian Spring began, Syrian activists used all available means of communication to demand freedom, democracy and their right to live in dignity. During the revolution, they have used these means to communicate attacks on civil populations and are now using these same means to publicize the grave health situation that they are facing, with a lack of hospitals, doctors and medicines. In this Letter, I report for the first time, how social media are being used by Doctor-Activists, as an alarm system of war diseases outbreaks, namely cutaneous leishmaniasis.

Old World cutaneous leishmaniasis (oriental sore), is produced by leishmanias belonging to the *Leishmania tropica* complex, when the bite of an infected sandfly *Phlebotomus papatasi,* or closely related species, liberates promastigotes into the skin [6,7]. Leishmaniasis is endemic in 98 countries on 5 continents. Syria is one of the most affected countries, with the highest estimated case counts, with 22,882 cases per year (up to the estimation between 2004–2008) [8], with the first occurrence of cutaneous leishmaniasis documented as early as 1745 [9]. The common local name is "Aleppo boil".

During January-March 2013, I collected from YouTube and Facebook, twenty videos, filmed by Doctor-Activists, describing cutaneous leishmaniasis outbreaks in different towns from Deir Ezzor Province (East Syria). I later confirmed these data by direct contact with local communities. Some of the affected towns are Al Mayadin (35°01'66"N,40°45'00"E), Al Muhassan (35°23'78"N,,40°31'90"E), Hajin (34°68'66"N,40°83'48"E), Diban (35°00'60"N,40°51'33"E), Al Shaafa (34°57'05"N,40°93'34"E), Al Baguz (34°43'19"N,40°98'77"E), Al Susa (34°54'69"N,41°29'77"E), Al Saial (34°35'34"N, 40°54'16"E), Abu-Hamam (34°85'61"N,40°67'16"E), Shoaitat (34°48'36"N, 40°41'41"E), Al Gordi (34°51'55"N, 40°38'15"E) and Swedan (34°89'55"N, 40°60'63"E). The prevalence of this disease reaches 25% of the population in some small towns (Figure 1). One of the Doctor-Activists tells that his group receives between 400 and 500 patients with cutaneous leishmaniasis every week in only one locality, Hajin Town. The situation seems to be critical in this town. For example in Abu Alghater School, there are 125 children affected from the total number of 450 students, and in Al-Mazraa there are 120 cases with cutaneous leishmaniasis from about 500 students.

**Figure 1:**
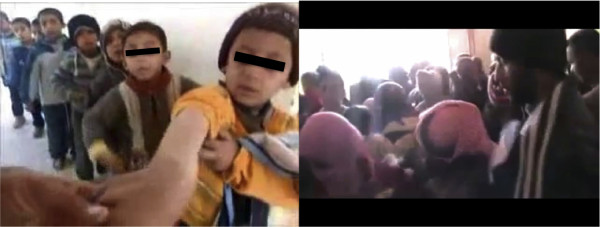
**Left: Queue of children waiting to be treated for leishmaniasis in Abu Alghater School.** Right: Mass of people waiting to be treated in Al Gordi Town, Deir Ezzor, Syria (reproduced with permission).

Lesions were reported on body areas exposed to the sandfly vectors (mainly face hands and feet), causing one or numerous sores on the skin. Initially the lesion is a small red papule up to 2 cm in diameter. Over time, papules become darker and form ulcers with central craters and raised edges. Ulcers can be moist and exude pus, or dry with a crusted scab [10] (Figure 2).

**Figure 2:**
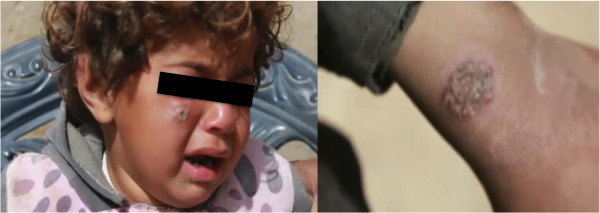
Lesions of cutaneous leishmaniasis in different body parts from schoolchildren, Deir Ezzor, Syria (reproduced with permission).

The Doctor-Activists in Deir Ezzor Province are treating cutaneous leishmaniasis by intralesional injection of meglumine antimoniate drugs, using an insulin syringe. For large lesions, injections are done in all surrounding parts to cover the wound surface completely (Figure 3). However, in such war situations, drugs are in limited supply. The Doctor-Activists Team in Al Gordi town stated that they have drugs sufficient to treat only two patients, while they have more than 600 affected with cutaneous leishmaniasis. WHO [11] recommends treating cutaneous leishmaniasis with pentavalent antimonial drugs (*e.g.* sodium stibogluconate or meglumine antimonate) at 20 mg/kg per day for 20–28 consecutive days. This means that nowadays, many affected with cutaneous leishmaniasis in Syria are not treated and the treated ones fail to complete their full course of treatment. Recently some drugs have been supplied by some charity organizations such as Inkad, Al Susa and Al Baguz.

**Figure 3:**
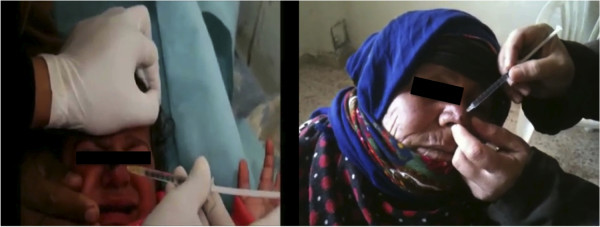
Intralesional injection of meglumine antimoniate drugs for cutaneous leishmaniasis treatment in (left) a schoolchild from Al Susa and (right) an old woman from Diban, Deir Ezzor, Syria (reproduced with permission).

Another approach to control this outbreak is the treatment of the sandfly vector by the use of insecticides. The local community of Al Susa Town is using such a tool, applying insecticides by variety of sprayers. The insecticide is being applied to treat focal areas such as waste deposits and abandoned houses. However we are not aware of which insecticide is being used (Figure 4).

**Figure 4:**
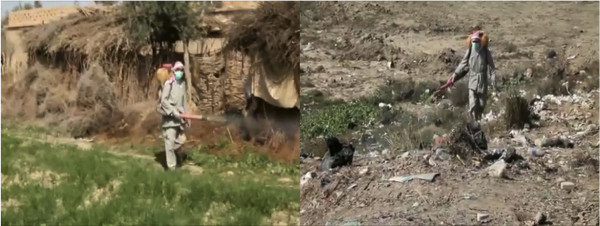
Insecticide application to treat the sandfly vector focal areas, such as waste deposits and abandoned houses, Al Susa Town, Deir Ezzor, Syria (reproduced with permission).

This letter highlights the positive effect of the social media as an alarm system used by Doctor-Activist to report war disease outbreaks. However, these social media are still far from describing the full epidemiological study or treatment efficacy, since they lack professional surveillance and vital records. They are, however, an efficient means to attract the attention of the international organizations, which should assume their responsibilities and play their part in controlling the outbreak of such diseases.

### Consent

Written informed consent was obtained from the patient for publication of this report and any accompanying images. For children images, written informed consent was obtained from the patient’s guardian/parent/next in keen for publication of this report and any accompanying images.

### Competing interests

The author declares that he has no competing interests.
